# Intestinal celiac disease - related autoantibodies

**DOI:** 10.3389/fimmu.2025.1567416

**Published:** 2025-06-06

**Authors:** Giorgia Fontana, Fabiana Ziberna, Egidio Barbi, Grazia Di Leo, Luigina De Leo

**Affiliations:** Institute for Maternal and Child Health, I.R.C.C.S. Burlo Garofolo, Trieste, Italy

**Keywords:** celiac disease, autoantibodies, tissue transglutaminase, intestinal celiac disease - related antibodies, diagnostic marker

## Abstract

Celiac disease is a widespread autoimmune enteropathy with a genetic predisposition triggered by gluten intake. The only available treatment is a strict lifelong gluten-free diet. The diagnosis is based on the detection of serum celiac disease - related antibodies and histopathological analysis of duodenal biopsies. However, celiac disease has a wide spectrum of clinical, histological, and serological manifestations, and in some patients, the diagnosis can be challenging. Celiac disease - related antibodies antibodies are produced by intestinal B cells and can be detected in the small intestinal mucosa before their appearance in serum or before mucosal damage. In this paper, we reviewed the literature concerning the diagnostic value of intestinal celiac disease - related antibodies.

## 1 Introduction

Celiac disease is a complex genetic autoimmune enteropathy triggered by dietary gluten in wheat, rye, and barley (1). This disease is one of the most widespread lifelong disorders with a reported prevalence of 1%–2% in the general population (2, 3). The only available treatment is adherence to a strict gluten-free diet. The clinical manifestations of celiac disease are broad and range from mild to severe. Patients may suffer from gastrointestinal symptoms (diarrhea, malabsorption, recurrent abdominal pain, and weight loss), and/or various extraintestinal manifestations (including osteoporosis, arthritis, dermatitis herpetiformis, and neurological, cardiac, and obstetric disorders), or even remain asymptomatic (4–12). The frequency and types of clinical manifestations of celiac disease can be significantly different across age groups. In adults, extraintestinal symptoms such as psychiatric problems, infertility, recurrent spontaneous abortion, and peripheral neuropathy are common. In contrast, children and adolescents experience abdominal discomfort more frequently (13, 14).

Moreover, celiac disease is often associated with other autoimmune diseases like dermatitis herpetiformis, type 1 diabetes (15), immunoglobulin type A (IgA) deficiencies, neuropathy, and gluten ataxia (16–18).

The disorder almost exclusively occurs in individuals carrying the human leukocyte antigen (HLA)-DQ2 and/or DQ8 haplotypes, indicating that the genetic susceptibility plays a pivotal role in the pathogenesis of celiac disease. However, HLA-DQ2 and/or DQ8 are carried by a third of the general non-celiac population; thus, other genetic and/or environmental factors are likely to be involved in the disease onset (19). Genome-wide association studies have identified 41 additional non-HLA loci associated with celiac disease. These genetic factors, which individually contribute little to the disease development, are involved in the regulation of various aspects of the immune system and barrier function and could modulate disease presentation and phenotype (20, 21).

In patients with celiac disease, the ingestion of gluten induces structural changes in the gut and contributes to the production of specific autoantibodies.

Small intestinal damage is characterized by villous atrophy with crypt hyperplasia and an increased number of intraepithelial lymphocytes (22). The grade of intestinal damage can be classified using the widely used Marsh–Oberhuber classification system, ranging from grade 0 (normal small intestinal mucosa) to grade 3c (total villous atrophy) (23). A correct histological evaluation requires proper handling and orientation of specimens to avoid artifacts; it also requires the collection of at least six biopsies (two from the duodenal bulb and four from the second or third portion of the duodenum) (24).

Celiac disease - related autoantibodies are widely used in clinical practice. They are detectable in serum samples and include anti-tissue transglutaminase, anti-endomysial, and anti-gliadin peptide antibodies. IgA anti-tissue transglutaminase (anti-ttg) antibodies are directed against the specific auto-antigen tissue transglutaminase and are recognized as being the most sensitive marker in the case of an active form of celiac disease. Anti-ttg antibodies can be detected using different methods such as enzyme-linked immunosorbent assay, chemiluminescence, or fluorescence immunoassay. The detection of IgA anti-ttg antibodies, together with the measurement of total serum IgA to exclude selective IgA deficiency, is performed as a first-level screening test (25, 26).

Serum IgA anti-endomysial autoantibodies (EMAs) are detected using an indirect immunofluorescence assay on tissue sections of monkey esophagus, human umbilical cord, or primate liver. EMAs recognize the same antigen as anti-ttg antibodies, from which they only differ in terms of detection method. EMA tests performed by immunofluorescence assay selectively and specifically detect anti-ttg antibodies that recognize celiac disease-related conformational epitopes. The specificity of IgA EMA is very high (approximately 100%); however, its detection requires operators who are skilled in the methodological procedure. Therefore, in the diagnostic work-up of celiac disease, the EMA assay is suitable as a confirmatory test and not as a first-level screening (27, 28).

Immunoglobulin type G (IgG) and IgA anti-deamidated gliadin peptide (anti-DGP) antibodies have a lower positive predictive value for the diagnosis of celiac disease. However, in children <2 years of age, the detection of anti-DGP antibodies, in addition to anti-ttg antibody determination, may increase the diagnostic sensitivity (17, 25, 29–31).

**Figure 1:**
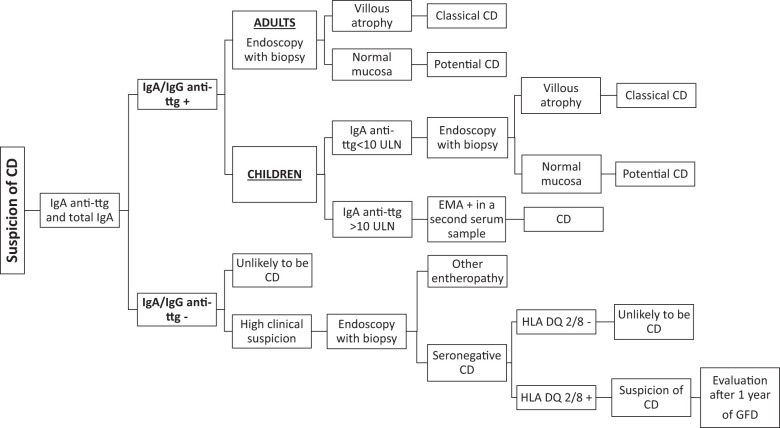
Diagnostic workflow for celiac disease. Anti-ttg, anti-tissue transglutaminase; IgA, immunoglobulin A; CD, celiac disease; GFD, gluten-free diet; ULN, upper limit of normal.

A high concentration of IgA anti-ttg antibodies correlates with severe mucosal damage. According to these findings, the European Society for Paediatric Gastroenterology, Hepatology and Nutrition guidelines have adopted the no-biopsy approach to diagnose celiac disease in pediatric patients with IgA anti-ttg antibodies positivity >10 times the upper limit of normal, confirmed by EMA finding in a second blood sample (26). This approach avoids endoscopy-related risks and costs in at least 50% of children with a suspicion of celiac disease (24, 32–34). In adults, the typical histological changes on the small intestinal mucosa, together with positive serologic markers (IgA anti-ttg antibodies), are required for a diagnosis of celiac disease (35, 36). Growing evidence suggests that a serology-based celiac disease diagnosis without biopsy could be applicable to adults as well as children (37, 38) (**Figure 1**).

In individuals with associated selective IgA deficiency (serum IgA < 7 mg/dL), testing for anti-ttg, EMAs, and anti-DGP antibodies in the IgG class is recommended (39–41).

In patients testing positive for serum celiac disease - related antibodies with intestinal villous atrophy, the diagnosis of classical celiac disease is clear-cut. However, the diagnosis can be challenging, and a significant proportion of celiac patients (10%–30%) remain uncertain and undiagnosed (42–45). Usually, these patients test positive for serological markers and show normal intestinal mucosa (potential celiac disease) (42, 46) or test negative for serological markers and show villous atrophy on small intestinal biopsy (seronegative celiac disease) (47). The complexity of these conditions requires careful and case-by-case evaluation. Almost a third of patients with potential celiac disease who continue to eat gluten develop overt celiac disease over time, whereas a similar proportion experience normalization of serology. Most symptomatic patients with potential celiac disease benefit from a gluten-free diet (48). In the presence of flat villi and negative serological celiac disease - related antibodies, seronegative celiac disease can be suspected, and genetic testing with detailed and complete HLA typing must always be performed. After excluding other seronegative enteropathies, the diagnosis of seronegative celiac disease can be established in symptomatic patients with a genetically compatible pattern, small intestinal damage, and negative serology. Both clinical and histological improvements after 1 year of a gluten-free diet are required to confirm the diagnosis (49) (**Figure 1**).

Serum celiac disease - related antibodies are produced by intestinal B cells. They can be detected in intestinal biopsy samples using *in vitro* diagnostic methods (e.g., anti-endomysium biopsy kit; Eurospital, Trieste, Italy) or research-type methods (e.g., phage-display libraries) in the early phases of the disease when the duodenal mucosa is still normal and serum celiac disease - related antibodies are negative or positive at a low titer (50–55). Therefore, intestinal EMAs and anti-ttg antibodies are specific and sensitive markers to identify patients with potential or seronegative celiac disease. In particular, in seronegative celiac disease, the specific autoantibodies have been detected in intestinal biopsy samples, suggesting that in these patients, the celiac disease autoimmune reaction may be confined to the gut (51). The aim of this review was to provide updated information about the role of celiac disease - related autoantibodies produced in the intestinal compartment.

## 2 Intestinal production of celiac disease-related antibodies

Gluten-derived gliadin peptides can enter the lamina propria and induce an immune response. Some gliadin peptides (e.g., p31–43 peptide) are toxic and induce epithelial stress and proinflammatory events, activating the adaptive immune response. Others (e.g., 33mer peptide) are immunotoxic and activate gluten-specific CD4+ T lymphocytes in the lamina propria of the small intestinal mucosa. The tissue transglutaminase enzyme modifies gluten-derived peptides through a deamidation reaction and increases their affinity to the pockets of the HLA-DQ2/DQ8 on the antigen-presenting cells. The presentation of the deamidated gluten peptides to T cells triggers the activation of the gluten-specific CD4+ T-helper 1 (TH1) cells, which start to secrete different proinflammatory cytokines such as interferon (IFN)-γ, interleukin (IL)-21, IL-15, IL-18, and type 1 interferons (56–59). According to the hapten-carrier hypothesis, gluten peptides can serve as a carrier when bound in a complex with ttg, thereby allowing tissue transglutaminase-specific B cells to receive activation help from gluten-specific, HLA-DQ-restricted CD4+ T cells. The activated B cells differentiate into plasma cells secreting IgA, mostly, and IgG antibodies against ttg and DGP. The gut is eventually the site of immunologic tolerance breakdown against the auto-antigen ttg (60).

Marzari et al. (50) demonstrated that celiac-related antibodies are produced by specifically activated intestinal B lymphocytes. The humoral response against ttg was investigated by means of a phage-display antibody library. This technique allows the display of antibody repertoires of a patient on the surface of phages that carry the encoded protein gene inside. Each phage expresses only one specific antibody. Mucosal phage-display antibody libraries were produced from both intestinal and peripheral B lymphocytes of three patients with celiac disease. Anti-ttg antibodies were isolated only from the intestinal lymphocyte libraries and not from the peripheral lymphocyte libraries. These results strongly suggest that celiac disease-related antibodies are synthesized locally in the intestine. The immune response production is predominantly IgA, partially IgM, and, in limited cases, IgG. These immunoglobulin antibodies are characterized by a restricted use of variable domains of heavy chain (VH) families, with a preferential usage of the VH5 family. Notwithstanding a chronic exposure to the antigen, celiac-specific antibodies display a low number of somatic mutations (50).

Di Niro et al. (61) found a high abundance of long-lived tissue transglutaminase-specific plasma cells in duodenum specimens from a celiac patient. After a gluten-free diet, these plasma cells decreased; however, they can be found in the gut even after several years of dietary treatment.

The study of Korponay-Szabó et al. (62) on duodenal frozen specimens demonstrated that anti-ttg antibodies are already deposited in the morphologically normal small intestinal mucosa before their appearance in the serum and before intestinal damage. Intestinal celiac disease - related antibodies show a typical pattern of recognition and specifically bind to the ttg in the jejunal sub-epithelium along the villous, in crypt basement membranes, and the connective tissue layer around the smooth muscle fibers of extraintestinal tissues.

## 3 Effects of intestinal celiac disease-related antibodies

The biological effects of celiac disease-related antibodies have been investigated in different *in vitro*/*in vivo* models. However, the role of these antibodies in the pathogenesis of celiac disease remains controversial (63–65).

Anti-ttg antibodies bind to tissue transglutaminase; thus, it is logical to investigate whether these antibodies can affect its enzymatic activity. Several studies have explored this hypothesis with quite contradictory results. Experiments have shown different effects of anti-ttg antibodies on ttg activity: inhibitory (66, 67), enhancing (68, 69), or none (61). Discordant observations could be related to different experimental and methodological approaches and also to the variability of polyclonal antibodies that recognize different epitopes and exert different effects on ttg enzymatic activity. Moreover, ttg is a widely distributed multifunctional protein involved in a broad range of cellular and metabolic functions carried out in a variety of cellular compartments. Therefore, the controversial biological effects of anti-ttg antibodies may depend on the multiple functions of the protein (70, 71).

Furthermore, anti-ttg antibodies may have biological effects on various cell types (63, 64). The effects of anti-ttg antibodies on the intestinal epithelial cells have been widely evaluated. Because of the inability of antibodies to penetrate the cell membrane, anti-ttg antibodies are more likely to function in the extracellular environment. *In vitro* experiments on intestinal epithelial cells showed that anti-ttg antibodies may induce cell proliferation and inhibit the differentiation of intestinal epithelial cells. Therefore, celiac disease - related antibodies could contribute to the development of crypt hyperplasia with the lack of differentiation and enhanced proliferation of the epithelium, typically observed in untreated celiac disease (72). Moreover, anti-ttg antibodies interact with the extracellular ttg and induce cytoskeleton reorganization with actin redistribution and permeability changes. In particular, anti-ttg antibodies are thought to increase the permeability of the epithelial barrier (73), allowing gliadin peptides to access the lamina propria and affecting epithelial cell biology (74, 75).

Celiac disease - related autoantibodies also affect the function of endothelial cells. Anti-ttg antibodies were found deposited around the small-bowel mucosal blood vessels (51, 62) and were able to inhibit several steps of angiogenesis in *in vitro* experiments (76). Thus, the anti-angiogenic effects of celiac disease - related autoantibodies could lead to the disorganization of the intestinal vascular network and the severe vessel immaturity observed in the small intestinal mucosa of untreated celiac patients (77).

In addition to affecting epithelial and endothelial cell biology, the celiac disease - related autoantibodies induce the activation of monocytes upon binding to toll-like receptor 4 (73). Although the role of monocytes in celiac disease is unclear, the activation of monocytes by celiac disease - related autoantibodies may be involved in the pathogenesis by attracting immune cells and guiding them to the inflamed tissue and activating matrix metalloproteinases (78, 79) through the secretion of inflammatory cytokines. These findings suggest that celiac disease - related autoantibodies may represent a link between the innate and adaptive immune response in the pathogenesis of celiac disease (73).

## 4 Detection of intestinal celiac disease-related antibodies

**Figure 2:**
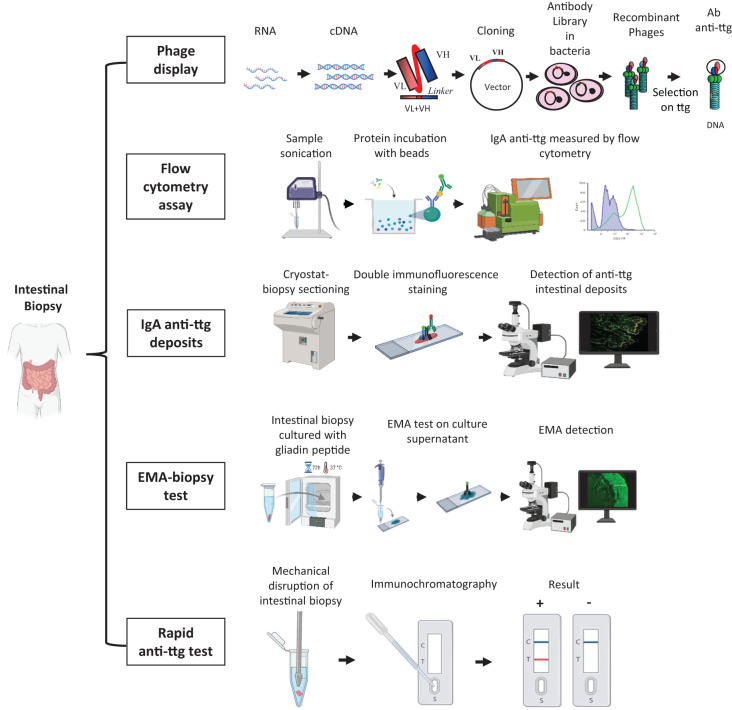
Different methods to detect and measure the intestinal celiac disease - related antibodies. *Phage-display library*: intestinal RNA is reverse transcribed into cDNA from which antibodies are amplified as single-chain fragment variable and cloned into a phagemid vector. Vectors are inserted into bacteria to generate the antibody library. Antibodies are exposed on recombinant phages and affinity-selected on tissue transglutaminase antigen. *Flow cytometry assay*: intestinal biopsy is sonicated, and intestinal IgA anti-ttg antibodies are acid-eluted. Proteins are incubated with beads coupled with human recombinant tissue transglutaminase and then with fluorescein-conjugated anti-human IgA to finally measure them using flow cytometry. *IgA anti-ttg deposits*: intestinal cryo-sections are stained with a fluorescein isothiocyanate anti-human IgA antibody and with an Alexa Fluor 594 anti-tissue transglutaminase antibody (double immunofluorescence staining). IgA anti-ttg deposits appear by fluorescence microscope analysis as yellow spots deriving from the colocalization of IgA (in green) and tissue transglutaminase (in red). *EMA biopsy test*: intestinal biopsies are cultured with gliadin peptide for 72 h at 37°C, and the culture supernatant is spotted on sections of monkey esophageal mucosa or human umbilical cord to detect EMA antibodies by fluorescence microscope analysis. *Rapid anti-ttg detection test*: an intestinal biopsy sample is mechanically disrupted in a saline buffer, which is loaded into an immunochromatographic cassette. Intestinal IgA/IgG/IgM anti-ttg antibodies recognize the tissue transglutaminase antigen absorbed onto membrane inside the cassette and appear as a red line along with a blue control line. EMA, anti-endomysium antibody; ttg, tissue transglutaminase; VH, variable domain of heavy chain; VL, variable domain of light chain. Created with BioRender.

Many technical approaches have been developed to detect and measure the intestinal celiac disease - related antibodies over time. These techniques include research-type (phage-display libraries, flow cytometry assay, and rapid anti-ttg detection test) and *in vitro* diagnostic methods (anti-ttg deposits and EMA biopsy). The characteristics of each technique are summarized and represented in **Table 1**; **Figure 2**. The diagnostic accuracy of this marker in patients already on a gluten-free diet has not been fully established. Therefore, the detection of intestinal celiac disease - related antibodies should be performed in patients on a gluten-containing diet undergoing gastrointestinal endoscopy for the diagnosis of celiac disease.

**Table 1:** Techniques to detect intestinal celiac disease-related antibodies.

Techniques	Characteristics			
Technique type	Sensitivity	Specificity	Advantages	Limits
Phage-Display Libraries	97-98%	100%	Sample storage	Highly-qualified personnel Too demanding protocol Time consuming Qualitative results
Flow cytometry assay	100%	87%	Sample storage	Trained personnel Laboratory equipment (sonicator, flow cytometer)
IgA anti-ttg deposits	73-100%	80-100%	Sample storage	Trained personnel Well-oriented tissue sample Laboratory equipment (cryostat, fluorescence microscope)
EMA-biopsy test	94-100%	99%	Easy handling assay Standardized kits Less time-consuming	Trained personnel Fresh sample
Rapid anti-ttg detection test	100%	97%	Quick and easy handling assay Feasible in any endoscopy unit	Fresh sample

The table summarizes characteristics of each technique.

IgA, immunoglobulin A; EMA, anti-endomysium antibody; ttg, tissue transglutaminase.

### 4.1 Phage-display libraries

According to this technique, a patient’s antibody repertoire is expressed and fused to a coat protein of a phage. The intestinal biopsy sample is stored in a reagent (e.g., TRIzol) to preserve RNA, which is reversely transcribed into cDNA. The antibodies are amplified, as single-chain fragment variable, from cDNA by polymerase chain reaction using a group of primers that identify all human V genes. Then, the antibodies are cloned into phagemid vectors, which are inserted into bacteria. Finally, antibodies are affinity-selected on the auto-antigen ttg.

It is possible to create a phage-display library for each type of immunoglobulin. Hence, in case of selective IgA deficiency, an IgG or IgM phage-display library can be produced and selected to isolate specific intestinal antibodies.

This technique offers the advantage of obtaining both the protein and the corresponding DNA sequence and is extremely sensitive and specific (80). Unfortunately, it is too sophisticated and laborious to be implemented in clinical practice (81).

### 4.2 Flow cytometry assay

Intestinal IgA anti-ttg antibodies are acid-eluted after sonication of intestinal biopsy fragments. Proteins are incubated with beads coupled with human recombinant tissue transglutaminase and then with fluorescein-conjugated anti-human IgA. IgA anti-ttg antibodies are measured by flow cytometry. This method, described by Quaglia et al. (82), showed high sensitivity (100%) but low specificity (87%).

### 4.3 IgA anti-ttg deposits

The direct double immunofluorescence technique allows the detection of intestinal IgA anti-ttg antibodies on unfixed duodenal frozen sections. After a multicolor fluorescence microscope analysis, intestinal IgA anti-ttg antibodies appear as yellow spots at a sub-epithelial level and around crypts. In patients with selective IgA deficiency, the lack of secretory IgA is replaced by a compensatory increase in secretory IgM. Therefore, in these patients, intestinal anti-ttg deposits are detected as IgM.

This technique has a sensitivity and a specificity ranging from 73% to 100% and from 80% to 100%, respectively (17, 26, 35, 83–86). In untreated celiac patients, the sensitivity is age-related: 100% in adults (87, 88), 96% to 100% in children (89, 90), and 73% in children younger than 2 years (85). This detection method is less demanding than a phage-display library. However, trained personnel and specific laboratory equipment such as a cryostat and a fluorescence microscope are required to perform this analysis. Therefore, this technology is not widely available.

### 4.4 EMA biopsy test

The EMA biopsy test is based on a commercially available kit (anti-endomysium biopsy; Eurospital, Trieste, Italy) and allows the detection of intestinal celiac disease - related antibodies as IgA EMA in the biopsy culture medium. In selective IgA deficiency, IgM EMAs are investigated. Briefly, intestinal biopsy is cultured for 72 h at 37°C in the presence of gliadin peptides. Then, EMA antibodies, secreted in the culture supernatants, are detected by indirect immunofluorescence on sections of monkey esophageal mucosa or human umbilical cord. Although this test is not too demanding, lab support is required (53, 54, 86, 91, 92). The diagnostic accuracy of the EMA biopsy test is similar to that of anti-ttg deposits and is even higher in patients with potential celiac disease. A comparison study between these two techniques in a pediatric population showed an extremely high agreement in both bulb and distal duodenum specimens. Moreover, this study identified the duodenal bulb as the site at which intestinal celiac disease antibodies have to be investigated (92).

### 4.5 Rapid anti-ttg detection test

A further evolution in the investigation of intestinal celiac autoantibodies is the rapid anti-ttg detection test. This test requires the mechanical lysis of intestinal biopsy in a buffer solution. The supernatant is loaded into an immunochromatographic cassette, and intestinal IgA/IgG/IgM anti-ttg antibodies specifically recognize the enzyme tissue transglutaminase absorbed onto a membrane inside the cassette. In case of a positive test, a red/pink line appears along with a blue control line. Recently, a monocentric pediatric study on a pediatric population described this smart test for the first time and revealed a high diagnostic accuracy (98.6%) with 100% sensitivity and 97% specificity. A comparative analysis showed a perfect concordance of the rapid anti-ttg detection test with the EMA biopsy test. Moreover, it was demonstrated that the rapid anti-ttg detection test is reliable in recognizing intestinal celiac autoantibodies and potential and seronegative celiac disease. This test confirmed a higher sensitivity in detecting intestinal celiac antibodies in duodenal bulb specimens. All types of immunoglobulins (IgA/IgG/IgM) are detected by this test, which can also be performed in patients with selective IgA deficiency (54). This test could be used for an easy and fast detection of intestinal celiac disease - related antibodies, with a diagnostic result already available at the end of the endoscopy session. Its implementation in clinical practice would allow a better understanding of the prognostic value of intestinal anti-ttg antibodies and help clinicians in cases of suspected celiac disease that are difficult to classify.

## 5 A future role of intestinal celiac disease-related antibodies in diagnostic work-up

The guidelines for the diagnosis of celiac disease include recommendations for the evaluation and management of patients with celiac disease (93). Celiac disease-related antibodies are detected in the serum for the initial screening of patients with suspicion of celiac disease. Intestinal biopsy is required in most patients to confirm the diagnosis. Currently, the detection of intestinal celiac disease - related antibodies is not included in the guidelines for the diagnosis of celiac disease in children and adults. The diagnosis of celiac disease can be challenging because the spectrum of clinical manifestations is broad, and the current diagnostic criteria are inadequate to identify the whole spectrum of celiac disease. In addition to the classical celiac disease are the following: the potential celiac disease, in which the serology is positive but the intestinal mucosa is normal, and the seronegative celiac disease, in which serology is negative but the intestinal mucosa is damaged. Thus, serology and histology are not always sufficient to confirm the diagnosis. The scenario is even more complicated because sometimes the serum anti-ttg antibody value is low, serum EMA is weakly positive, or serology tests are negative or fluctuating, but the patient suffers from typical celiac intestinal symptoms or only extraintestinal manifestations (25). In these cases, the invasive and expensive upper gastrointestinal endoscopy procedure is needed to collect intestinal biopsy specimens. However, the histological analysis can be useless in case of patchy intestinal damage, mild enteropathy, or normal intestinal mucosa. Therefore, laboratory and histology findings may be inconclusive. The search for non-invasive biomarkers is ongoing, and additional diagnostic tools, such as cytokine determination, can help identify untreated celiac disease. Interleukin-21 has been linked to an increased disease risk, and the serum levels of IL-21 appear higher in celiac disease compared to healthy subjects and seem to correlate with serum anti-ttg antibodies and mucosal damage (94). However, celiac disease is an intestinal disease, and it is fundamental to focus on small intestinal samples of patients with celiac disease and the cell types present therein. Novel high-throughput techniques are currently being applied to uncover pathogenic pathways that are altered in the small intestine of celiac disease, including bulk and single-cell transcriptomics, medium and high-throughput proteomics, and cytometry by time-of-flight. These emerging techniques should pave the way to novel biomarkers in the diagnostics and monitoring of celiac disease (95).

Intestinal celiac disease - related autoantibodies, which are primarily produced in the intestine before spilling over into the bloodstream, could be an additional diagnostic tool to solve the challenging cases of celiac disease. The diagnostic value of intestinal celiac autoantibodies has increased over time because it was shown that they can predict an early gluten response as well as the development of intestinal damage (81). Auricchio R et al. (42) observed that patients with clear anti-ttg deposits in the small intestinal mucosa had more than twice the chance of developing flat mucosa compared with those who had no deposits. In the last European Society for Paediatric Gastroenterology, Hepatology and Nutrition guidelines, the algorithm for the diagnosis of celiac disease invites clinicians to consider anti-ttg deposits as an additional test in suspected celiac patients with Marsh 0/1 (normal intestinal mucosa/increased intraepithelial lymphocytes) and positive serology (26). Unfortunately, anti-ttg deposit methodology requires specialized technicians, and it is not widely available. However, the rapid anti-ttg detection test is an effective diagnostic tool that should be carried out in any gastroenterology unit to recognize all the clinical manifestations of celiac disease.

Several studies have confirmed the high diagnostic accuracy of intestinal celiac disease - related autoantibodies in both childhood and adulthood (53, 80, 96). Therefore, the inclusion of this marker in the diagnostic work-up and the next guideline update for celiac disease should be strongly considered.

## 6 Gluten dependence of intestinal celiac disease - related antibodies

A gluten-free diet is the only effective treatment for celiac disease, which guarantees remission of the disease, including mucosal healing and normalization of celiac disease-related serology in the majority of cases. After diagnosis and starting treatment, periodic follow-up is recommended to monitor compliance with a gluten-free diet, provide education about the disease, and ensure social support. Patients on an exclusion diet usually improve or resolve symptoms during the first 6 months of treatment. However, the rigorous exclusion of gluten from the diet is challenging, and clinical and/or histological remission is incomplete in a substantial number of patients. Mucosal healing is an achievable goal in pediatric patients, while it could be incomplete or absent in adults, in which the intestine often fails to heal despite negative serology and the absence of symptoms (93). This lack of mucosal healing may be associated with increased risk of lymphoproliferative malignancy, bone diseases, and refractory celiac disease (97, 98). However, in both pediatric and adult patients on gluten-free diets, symptoms improve, and the serological level of antibodies decreases when gluten intake is avoided. For this reason, it is important to make a correct and definitive diagnosis before removing gluten from the diet (99). When the gluten is removed from the diet, the diagnosis of celiac disease can be confirmed after the reintroduction of gluten in the diet through a gluten challenge. During the gluten challenge, the clinicians evaluate symptoms, autoantibodies, and histopathology (100, 101). The monitoring of symptoms is crucial to adjust the dose or duration of the challenge. Over the years, many pediatric and adult studies have been conducted to understand how much gluten is needed to have reliable data from the celiac disease test, but they have not been fully elucidated yet (93, 102, 103). If the ingestion of gluten causes mild or no symptoms, it could be useful to increase the amount or the period of ingestion in order to increase the confidence of the celiac disease test, while in case of distress, the challenge could be shortened (100, 104).

Tosco et al. (91) performed a study on 129 celiac pediatric patients under treatment and showed that the titers of serum anti-ttg antibodies decreased in the first year of follow-up and finally disappeared after 2 years of a strict gluten-free diet. However, negative celiac serology does not always correlate with the recovery of the intestinal mucosa (98). One reason for the presence of a persistent villous atrophy could be given by advertent or inadvertent gluten intake (105). Concurrently, intestinal celiac disease - related antibodies remain positive for a long time during a gluten-free diet. This happens because tissue transglutaminase-specific plasma cells, even if reduced in number during a gluten-free diet, are still present in a considerable amount when compared to those in non-celiac patients and continue to produce intestinal antibodies. Their number increases during villous atrophy and decreases when on a gluten-free diet, but only after a long period of diet do these cells and the related intestinal antibodies disappear also in celiac subjects (91, 106). This suggests that intestinal celiac disease - related antibodies should be used as a confirmatory diagnostic marker when patients have already started a gluten-reduced diet before intestinal biopsy and refuse to revert to a normal diet.

Recently, the therapeutic horizon for celiac disease has expanded thanks to great advances in our understanding of the molecular and immunologic aspects of celiac disease. Innovative treatments are currently under investigation and include gluten sequestration and degradation (107, 108), gluten tolerance induction (109), tight junction modulators (110, 111), transglutaminase inhibitors (112), lymphocyte trafficking (113), and homing inhibitors (114, 115). These new therapies are expected to improve both patient outcomes and quality of life by reducing the burden of dietary restrictions. The integration of these new therapies requires careful consideration of efficacy and safety (116–121). For this purpose, the detection of intestinal celiac disease - related autoantibodies should be taken into consideration to monitor the immune response to these emerging therapies.

## 7 Discussion

Celiac disease is a genetically driven autoimmune condition characterized by both intestinal and extraintestinal symptoms. The development of this genetic gluten intolerance is related to the production of specific autoantibodies in the small intestinal mucosa. The pathogenetic role and the clinical relevance as diagnostic biomarkers of celiac disease - related autoantibodies are still under investigation. In this review, we have mainly highlighted the role of intestinal celiac disease - related autoantibodies for diagnostic purposes. As described, testing for the presence of intestinal celiac disease - related antibodies could be a useful tool in difficult cases of celiac disease, and evidence is emerging about their potential pathogenetic role. The detection of celiac disease - related autoantibodies in serum samples plays a crucial role in the diagnosis of celiac disease. However, there is an increasing percentage of patients in whom the recommended diagnostic work-up is inconclusive (e.g., asymptomatic potential or seronegative celiac disease).

In conclusion, in this review, we have collected data suggesting that the current diagnostic criteria, based on serology and conventional histology, are not completely adequate to promptly identify the whole spectrum of celiac disease. Thus, intestinal celiac antibodies should be involved in routine diagnostics and the next update of the guidelines.
